# Facultative mutualisms: A double‐edged sword for foundation species in the face of anthropogenic global change

**DOI:** 10.1002/ece3.7044

**Published:** 2020-12-04

**Authors:** Tjisse van der Heide, Christine Angelini, Jimmy de Fouw, Johan S. Eklöf

**Affiliations:** ^1^ Department of Coastal Systems Royal Netherlands Institute of Sea Research and Utrecht University Den Burg The Netherlands; ^2^ Conservation Ecology Group Groningen Institute for Evolutionary Life Sciences University of Groningen Groningen The Netherlands; ^3^ Department of Environmental Engineering Sciences Engineering School for Sustainable Infrastructure and the Environment University of Florida Gainesville FL USA; ^4^ Department of Aquatic Ecology & Environmental Biology Institute for Water and Wetland Research Radboud University Nijmegen The Netherlands; ^5^ Department of Ecology, Environment and Plant Sciences Stockholm University Stockholm Sweden

**Keywords:** alternative stable states, anthropogenic global change, bistability, establishment threshold, facultative mutualism, foundation species, positive feedback

## Abstract

Ecosystems worldwide depend on habitat‐forming foundation species that often facilitate themselves with increasing density and patch size, while also engaging in facultative mutualisms. Anthropogenic global change (e.g., climate change, eutrophication, overharvest, land‐use change), however, is causing rapid declines of foundation species‐structured ecosystems, often typified by sudden collapse. Although disruption of obligate mutualisms involving foundation species is known to precipitate collapse (e.g., coral bleaching), how facultative mutualisms (i.e., context‐dependent, nonbinding reciprocal interactions) affect ecosystem resilience is uncertain. Here, we synthesize recent advancements and combine these with model analyses supported by real‐world examples, to propose that facultative mutualisms may pose a double‐edged sword for foundation species. We suggest that by amplifying self‐facilitative feedbacks by foundation species, facultative mutualisms can increase foundation species’ resistance to stress from anthropogenic impact. Simultaneously, however, mutualism dependency can generate or exacerbate bistability, implying a potential for sudden collapse when the mutualism's buffering capacity is exceeded, while recovery requires conditions to improve beyond the initial collapse point (hysteresis). Thus, our work emphasizes the importance of acknowledging facultative mutualisms for conservation and restoration of foundation species‐structured ecosystems, but highlights the potential risk of relying on mutualisms in the face of global change. We argue that significant caveats remain regarding the determination of these feedbacks, and suggest empirical manipulation across stress gradients as a way forward to identify related nonlinear responses.

## Graphical abstract

Ecosystems worldwide depend on habitat‐forming foundation species that engage in facultative mutualisms. Global change, however, is causing rapid declines of foundation species‐structured ecosystems, often typified by sudden collapse. Here, we synthesize recent advancements and combine this with model analyses to propose that facultative mutualisms can increase foundation species resistance to global change stressors, but simultaneously increase the risk of sudden collapse by generating or exacerbating bistability.

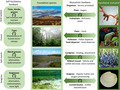

## 1 INTRODUCTION

Since the Industrial Revolution, humans have been altering environmental conditions at an unprecedented pace and scale (Kareiva et al., 2007; Steffen et al., 2018). Human‐induced global warming (Costanza et al., 1997; IPCC, 2014), together with more local impacts such as pollution, biotic invasions, overharvest, and land‐use changes, has triggered the sixth mass extinction of plants and animals (Cardinale et al., 2012). Biodiversity loss can be a direct consequence of such impacts, but can also arise from loss of organisms that are disproportionately important to ecosystem functions and structure (Angelini et al., 2011; Bruno et al., 2003; Estes et al., 2011). Particularly, the loss of foundation species (Dayton, 1972)—also known as autogenic ecosystem engineers (sensu Jones et al., 1997)—can elicit dramatic shifts in biodiversity and ecosystem functioning (Angelini et al., 2015; Borst et al., 2018; Bulleri et al., 2018; Ellison et al., 2005; van der Zee et al., 2016). Such spatially dominant habitat‐forming organisms—including trees, wetland plants, and reef‐building corals and bivalves—create complex 3‐dimensional biogenic structures that modulate the availability of critical resources and ameliorate physical stressors (Altieri et al., 2007; Donadi et al., 2013; Ellison et al., 2005; Hoegh‐Guldberg et al., 2007). Because many species are dependent on the presence of foundation species, disturbances that cause their decline often impact whole habitats to the extent that entire ecosystems and their associated communities collapse (Angelini et al., 2011; Bruno et al., 2003; Stachowicz, 2001).

Although the foundation species concept typically considers a single dominant species or a limited number of co‐occurring species in the same functional guild (e.g., as often occurs in forests, coral reefs, and macroalgae beds), many foundation species engage in obligate or facultative mutualisms (Angelini et al., 2016; de Fouw et al., 2016; Hay et al., 2004; Stachowicz, 2001). Obligate mutualisms, such as the association between fungi and phototrophs in lichens or the partnership between endosymbiotic zooxanthellae and corals, are by definition vital to both species irrespective of environmental conditions (Bronstein, 2015; Hoeksema & Bruna, 2000; Kiers et al., 2010). Facultative mutualisms, by contrast, are not vital to the organisms involved but can extend the natural environmental range limits of one or both organisms, thereby causing a species’ realized niche to exceed its fundamental niche (Afkhami et al., 2014; Bertness & Callaway, 1994; Bronstein, 2015; Bruno et al., 2003; Crotty & Bertness, 2015; Stachowicz, 2001). Mounting evidence suggests that facultative mutualisms commonly influence biodiversity and ecosystem structure, as many organisms are directly involved in networks of such beneficial interactions (Hay et al., 2004; Kiers et al., 2010; Silknetter et al., 2020; Stachowicz, 2001; Valdez et al., 2020).

**FIGURE 1:**
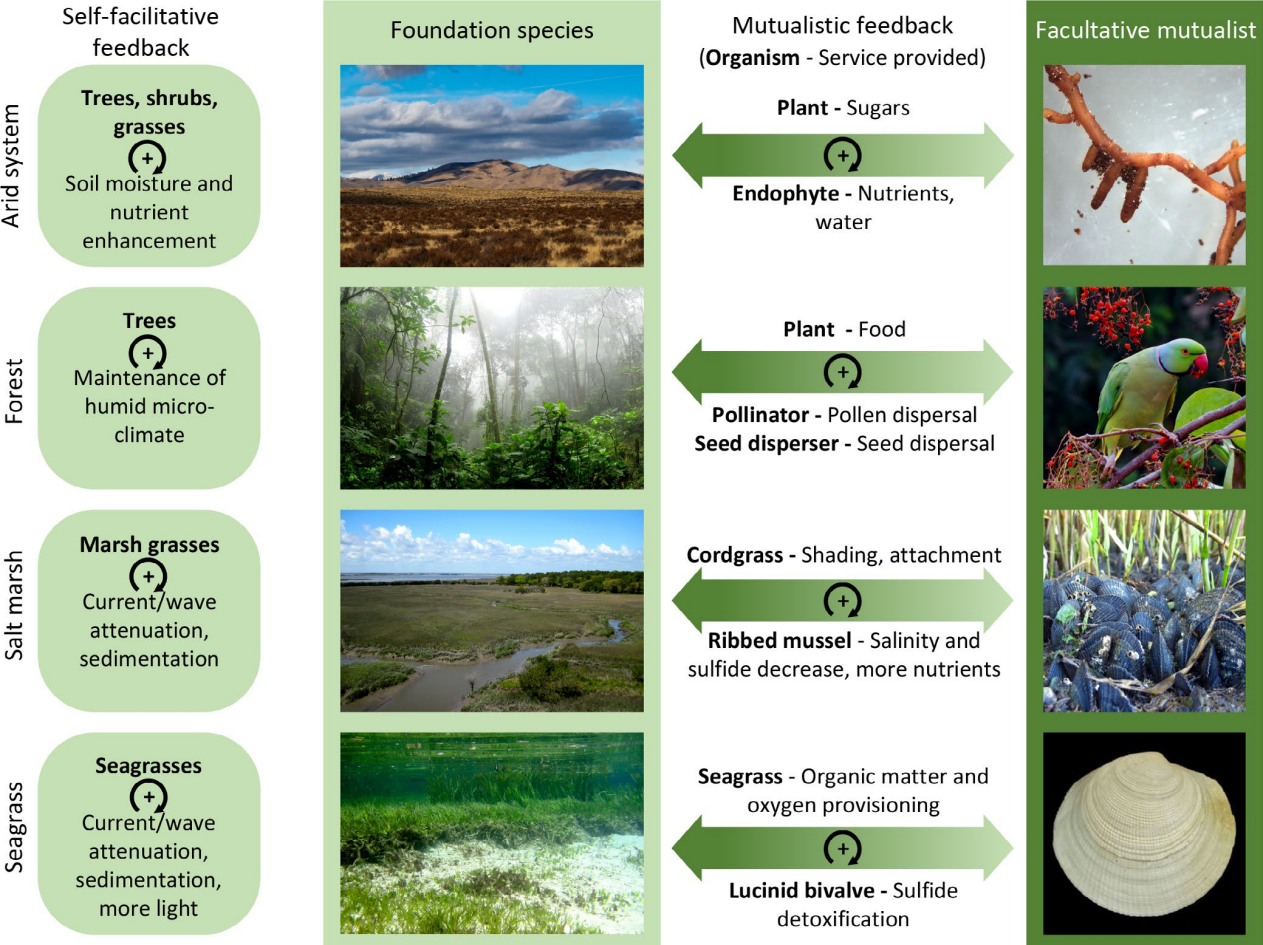
Four examples of ecosystems shaped by foundation species, their facultative mutualists, and the positive feedbacks generated

In this paper, we synthesize recent advancements to suggest that facultative mutualisms can strongly affect ecosystem stability and resilience when the interaction involves a foundation species. It is already well known that positive interactions in general, including mutualisms, support positive (also known as “exacerbating”) feedback mechanisms that, if strong enough, generate ecosystem thresholds or “tipping points” in environmental conditions beyond which ecosystems shift to alternative stable states (Kéfi et al., 2016; Maxwell et al., 2017). However, while studies have mostly focused on a single feedback mechanism, many ecosystems are characterized by multiple, potentially interacting feedbacks (Maxwell et al., 2017; van de Leemput et al., 2018). Here, we propose that facultative mutualisms and the feedbacks they initiate can increase foundation species’ resistance to human‐mediated global change stressors, but simultaneously predispose foundation species to abrupt collapse. To test this hypothesis, we build a conceptual framework that considers (1) how habitat modification by foundation species can lead to self‐facilitation via a positive feedback and consequently affect ecosystem resilience, and (2) how mutualisms generate another positive feedback that may interact with the first feedback. Finally, we present examples (Figure 1; Table 1) and discuss implications and future challenges.

**TABLE 1:** Examples of terrestrial, freshwater, and marine ecosystems structured by foundation species that generate a self‐facilitative feedback and can engage in a facultative mutualistic feedback

	Ecosystem	Foundation species	Facultative mutualist	Self‐facilitative feedback	Mutualistic feedback	Key references
Terrestrial	(semi‐)Arid systems	Trees, shrubs, grasses	Fungal endophytes	Plants enhance soil moisture with increasing density and patch size	Plants provide sugars; endophytes provide water and nutrients	Smith and Read (1997); Afkhami et al. (2014); Peay (2016)
	Temperate arid systems	Shrubs	Shrubs	Plants enhance soil moisture and soil nutrients with increasing density and patch size	Shrub mutualists provide predation shelter; further improve soil nutrient availability	Rietkerk et al. (2004); Tirado et al. (2015)
	Tropical forests	Trees	Ants	Dense tree canopy maintains humid microclimate	Acacia trees provide shelter and food; ants provide pollination, seed dispersal and defense against herbivores	Janzen (1966); Speight et al. (1999); Hirota et al. (2011); Zemp et al. (2017)
	Tropical forests	Trees	Pollinators and seed dispersers	Dense tree canopy maintains humid microclimate	Trees provide food and shelter; dispersers provide pollination and seed dispersal	Hirota et al. (2011); Peres et al. (2016); Zemp et al. (2017)
	Temperature montane forests	Trees	Seed dispersers	Tree canopies retain warm air, reduce wind stress, reduce evaporative loss of soil moisture and stabilize soils with increasing tree island size	Birds cache seeds near trees to facilitate the formation of tree islands that feedback to enhance tree island size and resilience	Malanson et al. (2007); Rodriguez‐Cabal et al. (2007); Pyatt et al. (2016)
Freshwater	Sphagnum peat bogs	Sphagnum mosses	Methanotrophic bacteria; N_2_‐fixing bacteria	Sphagnum mosses create wetland conditions above groundwater level by retaining and acidifying rainwater	Sphagnum mosses provide habitat in hyaline cells; bacteria oxidize CH_4_ to CO_2_ that mosses use for photosynthesis, fix N_2_ to alleviate N‐limitation	Raghoebarsing et al. (2006); Larmola et al. (2014)
	Helophyte swamps	Phragmites australis	Endophytic mycorrhizae	Dense Phragmites stands exclude grazing by waterfowl	Plants provide sugars; endophytes provide water and nutrients	Oliveira et al. (2001); Ernst et al. (2003); Reijers, Cruijsen, et al. (2019)
	Riparian forests	Trees	Fish	Dense forests attenuate flow, trap and stabilize sediment during inundation	Trees provide fruits as food; fish provide seed dispersal	Horn (1997); Horn et al. (2011); Silknetter et al. (2020)
	Shallow lakes	Submerged freshwater macrophytes	Mesograzers	Submerged macrophytes attenuate hydrodynamics, trap sediment and improve light conditions	Submerged macrophytes provide predation shelter; mesograzers consume epiphytic algae growing on plant leaves	Scheffer (1999)
Coastal/Marine	Seagrass meadows	Seagrasses	Mesograzers	Dense seagrasses attenuate hydrodynamics, trap sediment and improve light conditions	Seagrasses provide predation shelter; mesograzers consume epiphytic algae growing on plant leaves	Valentine and Duffy (2007); Maxwell et al. (2017)
	Tropical seagrass meadows	Seagrasses	Coraline algae	Dense seagrasses attenuate hydrodynamics, trap sediment and improve light conditions	Seagrasses protect the algae from removal by currents and waves; spiny coralline algae structures protect seagrass from grazing.	Maxwell et al. (2017); Leemans et al. (2020)
	Warm temperate to tropical seagrass meadows	Seagrasses	Lucinid bivalves	Dense seagrasses attenuate hydrodynamics, trap sediment and improve light conditions	Seagrasses provide organic matter for sulfide production and oxygen for sulfide oxidation; lucinids detoxify sulfides	van der Heide et al. (2012); de Fouw et al. (2016); de Fouw et al. (2018)
	Salt marshes	Marsh grasses	Ribbed mussels	Marsh grasses attenuate hydrodynamics, trap sediment with increasing density and patch size	Grasses provide shading and attachment; mussels lower salinity and sulfides, increase nutrients	Temmerman et al. (2007); Angelini et al. (2016); Derksen‐Hooijberg et al. (2019)
	Coastal dunes	Dune grasses	Fungal endophytes	Dune grasses trap eolian sand to form dunes in order to escape stress from seawater flooding	Plants provide sugars; endophytes provide water and nutrients	Kowalchuk et al. (2002); Reijers, Siteur, et al. (2019)
	Mangrove forests	Mangrove trees	Sponges	Mangroves attenuate hydrodynamics and trap sediments	Mangroves provide habitat, with roots as attachment substrate; sponges increase nutrient availability	Ellison et al. (1996); Huxham et al. (2010)
	Coral reefs	Hard corals	Herbivores	Corals form reefs that attenuate hydrodynamics, and serve as for attachment for recruits	Coral provide predation shelter; herbivores lower competition from macroalgae	van de Leemput et al. (2016)

## 2 FOUNDATION SPECIES AND SELF‐FACILITATIVE FEEDBACKS

**FIGURE 2:**
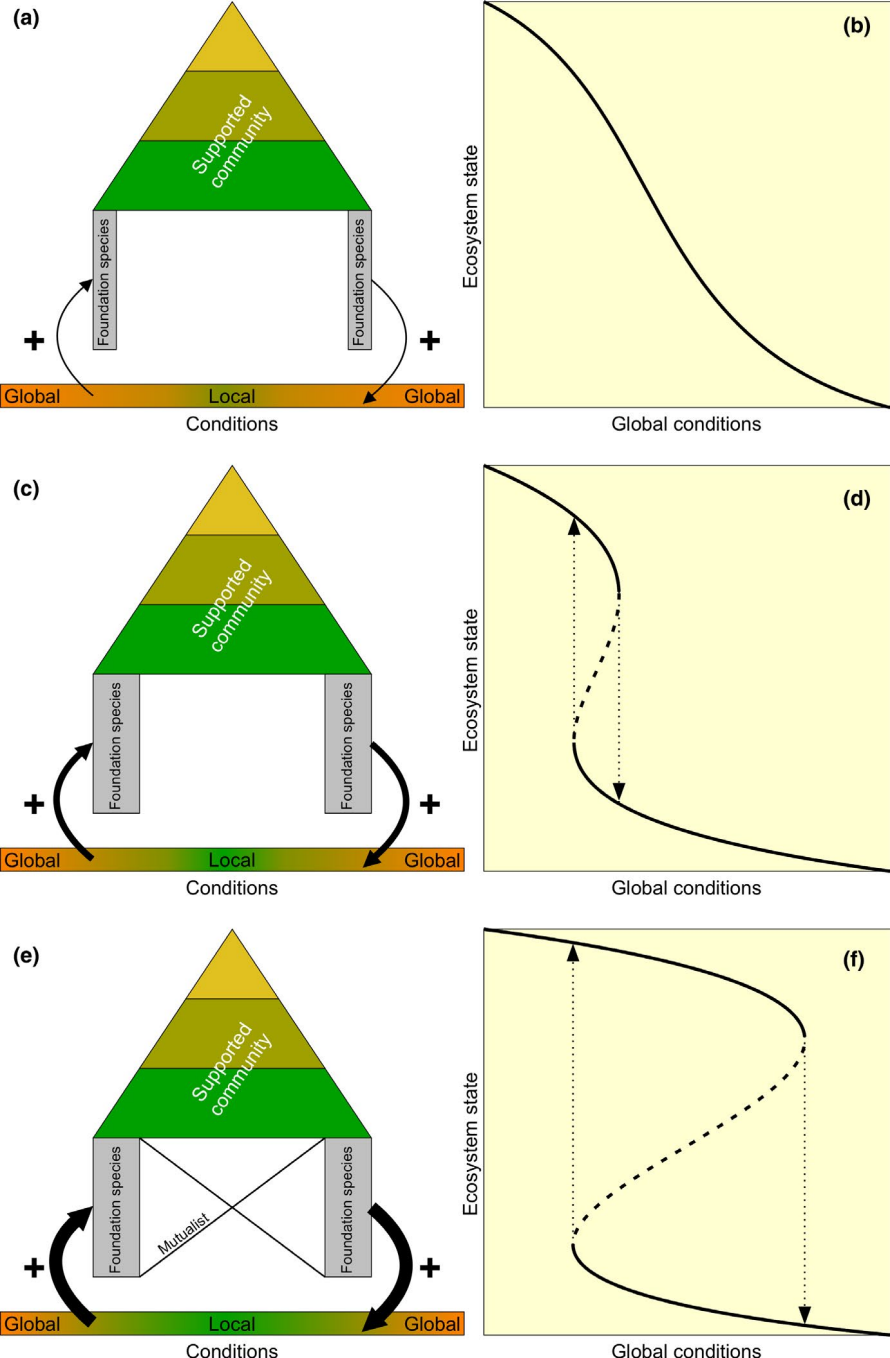
Self‐facilitative and mutualistic feedbacks stimulate foundation species and their associated community. When the maximum foundation species population size is low, beneficial modifications of local conditions (orange to green) are minor, implying a relatively weak self‐facilitative feedback (black arrows) (a), yielding a slightly nonlinear ecosystem response to changing global, ecosystem‐level conditions (b). A higher maximum population size generates a stronger feedback (c), thereby increasing the nonlinearity of the ecosystem's response to change and enhancing the potential for bistability (d). When the foundation species engages in a mutualism, both feedbacks act together to amplify environmental modifications (e), and the nonlinearity of the ecosystem's response to changing global conditions (f)

Foundation species modify the physical environment through their formation of complex physical structures that alter water and/or airflow, mediate nutrient cycling, and trap debris and detritus (Angelini et al., 2011; Dayton, 1972; Jones et al., 1994; Stachowicz, 2001). Although the typically positive consequences of such habitat modification for other community members have been the conceptual focus of many studies, foundation species also commonly improve living conditions for themselves and their conspecifics through the same mechanisms (Figure 2a–d) (e.g., van Hirota et al., 2011; de Koppel et al., 2005; Maxwell et al., 2017; Scheffer et al., 2012). Often, such self‐facilitation is generated via positive density dependence (Bertness & Callaway, 1994; Bruno et al., 2003) yielding a positive feedback, in which habitat quality improves with the density and/or patch size of the foundation species. Importantly, the strength and relevance of such self‐facilitation depends on environmental conditions. Changes made to an already suitable habitat via self‐facilitation will yield little overall improvement in living conditions. By contrast, self‐facilitation can be essential to a foundation species’ survival, growth, and reproduction in hostile conditions, by alleviating physical or biotic stress and thereby extending the foundation species’ own realized niche (Bruno et al., 2003; Crotty et al., 2018; He & Bertness, 2014). Examples of ecosystems where foundation species benefit from positive density dependence include tropical forest and desert vegetation that mediate water availability by creating a humid microclimate to stimulate plant growth (Hirota et al., 2011; Rietkerk et al., 2004); coral and shellfish reefs that facilitate settlement of additional coral and shellfish recruits by providing hard structures (Schulte et al., 2009); and seagrasses, salt marsh plants, and mangroves that enhance their own growth by stabilizing sediments, and trapping suspended particles to locally enhance nutrient availability (Balke et al., 2011; Zemp et al., 2017) (see Table 1 for further examples).

Many ecosystems structured by foundation species, including salt marshes, seagrass meadows, coral reefs, peatlands, and forests, have been rapidly declining, with losses often characterized by sudden collapse and low restoration success rates of degraded habitats (Ellison et al., 2005; Hoegh‐Guldberg et al., 2007; Maxwell et al., 2017; Rietkerk et al., 2004). A growing body of theoretical and empirical studies suggests that collapses are a consequence of the existence of feedbacks often derived from strong self‐facilitation (van de Koppel et al., 1997; Nyström et al., 2000; Scheffer et al., 2001). Ecosystems with such feedbacks typically respond in a nonlinear fashion to environmental change whereby the feedbacks buffer increasing external stress to support the foundation species’ persistence until a stress threshold is exceeded, at which point the foundation species experiences mass mortality. Moreover, if the feedback is sufficiently strong, it can cause alternative stable states (bistability); a condition where, depending on the initial state, either a foundation species‐structured or an alternative state is stable under the same environmental conditions (Figure 2a–d) (Scheffer et al., 2001). An important consequence is that recovery is very difficult once the foundation species’ abundance drops below the critical threshold required to induce the level of habitat modification needed to initiate and sustain new growth (Balke et al., 2011; Scheffer & Carpenter, 2003; Scheffer et al., 2001).

Over the last decades, there has been a surge of theoretical work on how feedbacks may lead to bistability and ecosystem collapse, as well as on indicators to detect nearness to collapse (e.g., Dakos et al., 2015; Scheffer et al., 2001). However, despite theoretical advancements, it remains difficult to predict these phenomena in the real world due to lack of knowledge on existing feedbacks or their strength and importance under prevailing conditions. As a consequence, density‐dependent positive feedbacks have yet to be systematically integrated into ecosystem management designs, and both the protection and restoration of foundation species‐dominated ecosystems remain extremely difficult (Bruno et al., 2003; Fischman et al., 2019; Silliman et al., 2015; Temmink et al., 2020). Moreover, contemporary studies have largely focused on a single feedback, often self‐facilitation, as the central mechanism underpinning nonlinear ecosystem responses and bistability (van de Leemput et al., 2016; Maxwell et al., 2017). In reality, however, foundation species‐dominated systems are often governed by multiple feedbacks, which may theoretically interact to alter nonlinear responses to environmental change (van de Leemput et al., 2016; Maxwell et al., 2017).

## 3 FOUNDATION SPECIES, SELF‐FACILITATIVE, AND MUTUALISTIC FEEDBACKS: A THEORETICAL FRAMEWORK

Mutualisms, by their very nature of providing reciprocal benefits, generate a positive feedback in which each partner stimulates the growth or survival of the other, thereby indirectly facilitating itself (Bronstein, 2015; Kiers et al., 2010). Because facultative mutualisms typically vary in strength with environmental conditions (Bronstein, 1994, 2015; Hoeksema & Bruna, 2000; Stachowicz, 2001), such interactions may invoke nonlinear responses of partnering species to environmental change, similar to the self‐facilitation by foundation species discussed above (Dakos & Bascompte, 2014; de Fouw et al., 2016, 2018; Lever et al., 2014; Maxwell et al., 2017). Indeed, theoretical work suggests that strong mutualistic interactions in plant–pollinator networks can cause bistability due to thresholds in environmental conditions, beyond which these mutualistic networks collapse (Dakos & Bascompte, 2014; Dean, 1983; Goh, 1979; Lever et al., 2014).

When a foundation species that, on the one hand, facilitates itself also engages in a mutualism, an inherent consequence is that the growth or survival of the foundation species is now mediated by two feedback mechanisms, not one (de Fouw et al., 2018; Maxwell et al., 2017). As the two feedbacks are both positive in nature, they may act in concert to facilitate the foundation species, potentially amplifying nonlinear ecosystem responses to environmental changes (Figure 2e–f). However, the two feedbacks may alleviate the same or different stressors, generating a context dependence that could strongly affect the foundation species’ vulnerability to anthropogenic global change.

To explore how the self‐facilitative and mutualistic feedbacks may interactively affect the resilience of foundation species‐structured ecosystems, we used a minimal mathematical model to investigate three scenarios: (1) The foundation species generates a single, self‐facilitative feedback that mitigates an environmental stressor; (2) the foundation species also engages in a facultative mutualism that mitigates a second environmental stressor; or (3) the foundation species also engages in a mutualism that acts on *the same* environmental stressor as the self‐facilitative feedback. Note that we define “stressor” as any external environmental force that can reduce the health of the foundation species (sensu Stachowicz, 2001).

The model consists of a system of two differential equations (de Fouw et al., 2018). The change in foundation species biomass or population size (*FS*) over time is described by the following differential equation:

(1) $$\frac{\mathrm{dFS}}{\mathrm{dt}}=g_{\mathrm{fs}}\cdot \left(1‐\frac{\mathrm{FS}}{K_{\mathrm{fs}}}\right)\cdot \mathrm{FS}‐m_{\mathrm{fs}}\cdot \mathrm{fS}1\cdot \mathrm{FS}‐m_{\mathrm{fs}}\cdot \mathrm{fS}2\cdot \mathrm{FS}$$

where *g*_fs_ is the maximum relative growth rate, *K*_fs_ is the carrying capacity, *m*_fs_ is the maximum relative mortality, and fS1 and fS2 are functions controlling the mortality due to stressors 1 and 2, respectively.

Following de Fouw et al. (2018), and as a conservative approach to the effect of the mutualist relative to logistic growth, we assume simple linear growth of the mutualist population size (*M*) that is facilitated by the foundation species:

(2) $$\frac{\mathrm{dM}}{\mathrm{dt}}=g_{m}\cdot \frac{\mathrm{FS}}{H_{\mathrm{fsm}}+\mathrm{FS}}\cdot \left(1‐\frac{M}{K_{m}}\right)‐m_{m}\cdot M$$

with *g*_m_ as the maximum growth rate, *H*_fsm_ as the half‐saturation constant for the positive effect of FS on *M*, *K*_m_ as the carrying capacity of *M*, and *m*_m_ as the relative mortality constant of *M*.

Function fS1 is described as follows:

(3) $$\mathrm{fS}1=S1\cdot \frac{H_{\mathrm{fs}1}}{H_{\mathrm{fs}1}+\mathrm{FS}}\cdot \mathrm{fM}1$$

where *S*1 is the maximum (i.e., when not mitigated) stress level from stressor 1, *H*_fs1_ is the half‐rate constant for reducing the stressor by the foundation species itself (i.e., the self‐facilitation effect), and fM1 is a function controlling the effect of the mutualist on stressor 1.

Function fM1 is described as follows:

(4.1) $$\mathrm{fM}1=\frac{H_{m1}}{H_{m1}+M}\text{if}\;\text{mutualist}\;M\;\text{is}\;\text{present}$$

(4.2) fM1=1ifmutualistMisabsent

in which *H_m_*_1_ is the half‐saturation constant for the effect of the mutualist on reducing stressor 1.

Finally, function fS2 is described as follows:

(5) fS2=S2·fM2

where *S*2 is the maximum stress level from stressor 2, and fM2 is the function controlling the mutualist's effect on stressor 2 (which is not mitigated by the foundation species):

(6.1) $$\text{fM}2=\frac{H_{m2}}{H_{m2}+M}\text{if}\;\text{mutualist}\;M\text{is}\;\mathrm{present}$$

(6.2) fM2=1ifmutualistMisabsent

in which *H_m_*_2_ is the half‐saturation constant for the reducing effect of the mutualist on stressor 2. Default model parameter settings are presented in Table 2. Scenario 1 was simulated with both fM1 and fM2 set at 1 (Equations 4.2 and 6.2, respectively); scenario 2 with fM1 at Equation 4.2 and fM2 at Equation (6.1); scenario 3 with fM1 set at Equation (4.1) and fM2 at Equation (6.2).

**TABLE 2:** Variables and default parameter settings of the conceptual model

	Default	Description
Variables		
*FS*	–	Foundation species population size
*M*	–	Mutualist population size
Parameters		
*g_fs_*	0.1	Maximum relative growth rate of the foundation species
*K_fs_*	1	Carrying capacity of the foundation species
*m_fs_*	0.3	Maximum relative mortality of the foundation species
*g_m_*	0.1	Maximum growth rate of the mutualist
*H_fsm_*	0.3	Half‐saturation constant for the positive effect of *FS* on *M*
*K_m_*	1	Carrying capacity of the mutualist
*m_m_*	0.05	Relative mortality constant of the mutualist
*S1*	0.05	Maximum (i.e., when not mitigated) stress level from stressor 1
*H_fs1_*	0.3	Half‐rate constant for the reducing effect of *FS* on stressor 1
*H_m1_*	0.3	Half‐rate constant for the reducing effect of *M* on stressor 1
*S2*	0.05	Maximum (i.e., when not mitigated) stress level from stressor 2
*H_m2_*	0.3	Half‐rate constant for the reducing effect of *M* on stressor 2

In each scenario, we used bifurcation analyses to evaluate the stability of the equilibria of the model at varying settings of stressors 1 and 2, and as a means of generally exploring how gradients in both stressors affect ecosystem resilience. For each analysis, the maximum stress level of either stressor 1 (*S*1) or 2 (*S*2) was increased in small steps, after which the model was run to stabilize to its equilibrium. This analysis was then performed backwards, such that each stressor was decreased in small steps. Finally, the two analyses were combined to construct bifurcation plots demonstrating how the foundation species’ population size varies across gradients in stressors 1 and 2 under each of the three scenarios. We determined unstable equilibria making a quasi‐steady‐state assumption and plotting equilibria for different values of the control parameters in GRIND for MATLAB.

## 4 MODEL RESULTS

**FIGURE 3:**
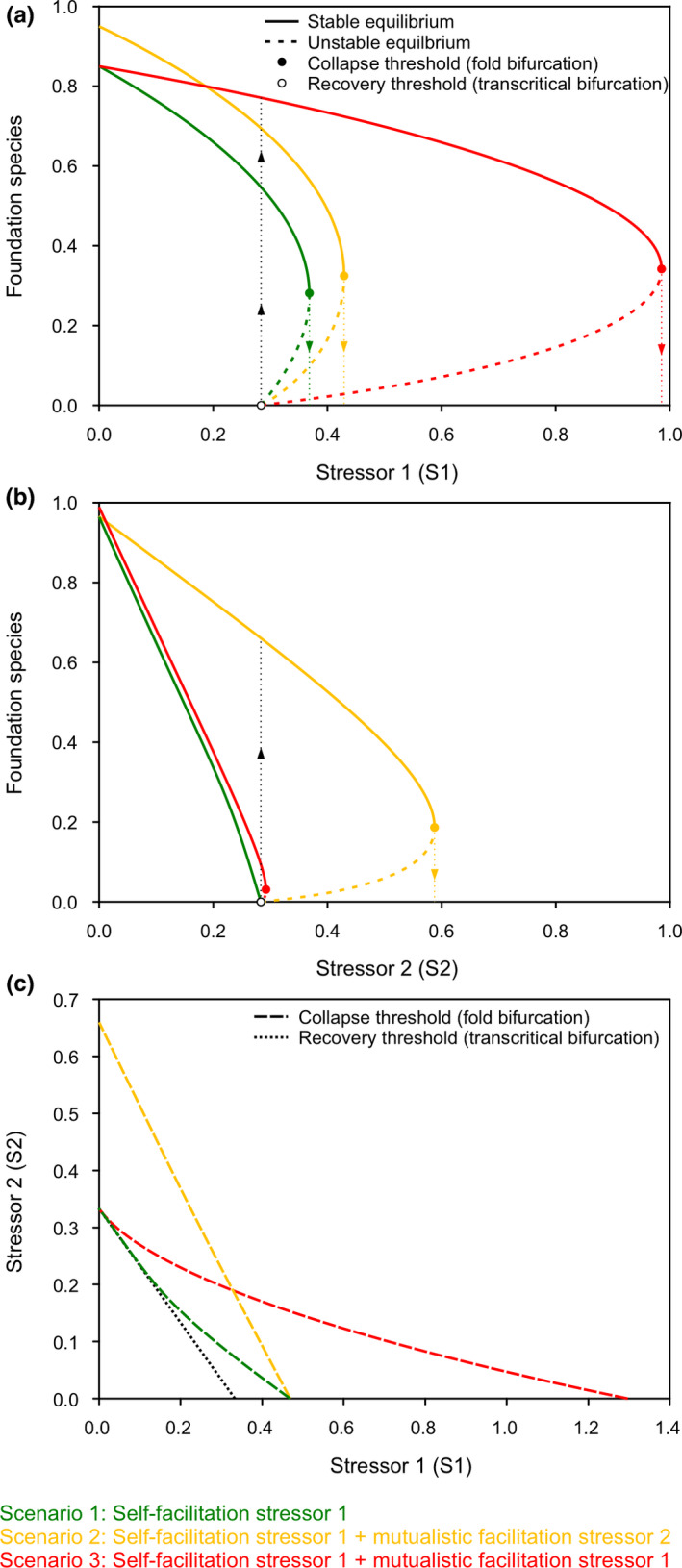
Bifurcation analyses of a minimal model of foundation species with mutualisms. The self‐facilitative feedback acts on stressor 1, generating bistability (scenario 1). The mutualism increases this bistability range, particularly when it also acts on stressor 1 (scenario 3), but even when mitigating stressor 2 (scenario 2) (a, c). When mitigating stressor 2, the mutualist also introduces bistability for this variable (b, c)

Similar to earlier studies of self‐facilitation (van der Heide et al., 2007; Scheffer & Carpenter, 2003; Scheffer et al., 2001), the model first predicts that self‐facilitation by the foundation species causes nonlinear behavior and bistability across the environmental stress gradient (Figure 3a). Second, when a mutualism that mitigates a second stressor is added, the foundation species’ overall health is enhanced (i.e., its net growth:mortality ratio is higher), allowing it to reach a higher maximum population size, and to occur across a broader range of both stressors (Figure 3b,c). However, nonlinearity also increases, such that bistability emerges for stressor 2, and the range of bistability increases for stressor 1. Third and finally, when the self‐facilitative and mutualistic feedbacks mitigate the same environmental stressor, they together amplify the buffering capacity for stressor 1, but also greatly enhance the bistability range (Figure 3a,c).

Although theoretical, this exercise yields several notable insights. First, foundation species can, by engaging in a mutualism, significantly expand their environmental range limit for a stressor (Afkhami et al., 2014). Interestingly, this “niche‐broadening” may be achieved even if the mutualism does not directly mitigate the stressor itself, but instead stimulates the foundation species by alleviating a second stressor. In addition to increasing ecosystem resistance to stress, the mutualism extends the range of hysteresis, amplifying nonlinear system responses to environmental stress. Consequently, environmental conditions may have to be improved over a much larger range to achieve natural recovery to a stable alternate state compared with systems whose behavior is not mediated by a mutualism. Finally, in binding both species to a common fate under conditions where the mutualism is essential for persistence, mutualistic interactions can increase the foundation species’ vulnerability to perturbations that affect the mutualist.

## 5 EXAMPLES FROM REAL ECOSYSTEMS

Foundation species in marine, aquatic, and terrestrial ecosystems often engage in mutualistic interactions (Figure 1; Table 1) (Hay et al., 2004; Stachowicz, 2001). For example, the vast majority of terrestrial plants engage in mycorrhizal or plant–pollinator interactions (Potts et al., 2010; Smith & Read, 1997), submerged marine and freshwater macrophytes provide shelter to grazers of algae that compete with the plants for light and nutrients (e.g., Peterson & Heck, 2001; Scheffer, 1999; Valentine & Duffy, 2007), *Sphagnum* mosses harbor methanotrophic and nitrogen‐fixing bacteria that increase CO_2_ and nitrogen availability to the plant (Larmola et al., 2014; Raghoebarsing et al., 2006), and sponges growing on the solid substrate provided by mangrove roots increase nutrient availability for the trees (Ellison et al., 1996). Here, we discuss four relatively well‐studied examples (Figure 1) in more detail to illustrate how both self‐facilitative and mutualistic feedbacks can affect ecosystem stability, and how human‐mediated environmental changes may affect these interactions.

### 5.1 Arid ecosystems

In arid systems, grasses and shrubs often modify soil conditions to their own benefit (Angelini et al., 2011; Kefi et al., 2007; Rietkerk et al., 2004; Rietkerk & van de Koppel, 2008). Following scenario 1, patches of grasses and shrubs enhance water availability by increasing infiltration with their root system, while simultaneously lowering evaporation through shading with increasing density and patch size (Klausmeier, 1999; Hille Ris Lambers et al., 2001; Rietkerk et al., 2002).

In many cases, these foundational plants engage in mutualistic interactions with mycorrhizal endophytes that benefit from the plants by receiving carbohydrates (Smith & Read, 1997). In return, these fungal mutualists can increase the productivity, biomass, and environmental range limits of the plants that adopt them by alleviating multiple stressors, including nutrient deficiency, salinity, and temperature stress (Millar & Bennett, 2016). In dry environments, plants can particularly benefit from mycorrhizae as they increase their tolerance to drought by increasing both water and nutrient uptake potential (Afkhami et al., 2014; Bahadur et al., 2019; Márquez et al., 2007; Peay, 2016). Such mitigation of drought and nutrient stress by both self‐facilitation and mutualism is similar to scenario 3, where the mutualist mitigates the same stressor (or two interrelated stressors in this case) as the foundation species (Figure 3).

Although mycorrhizae can mitigate abiotic stressors, excessive stress in the form of anthropogenic nutrient input or extreme drought can reduce the plants’ carbon allocation to the mycorrhizae (Millar & Bennett, 2016). Reciprocally, mycorrhizal partners have been found to adopt resource‐hoarding strategies under enhanced nutrient availability (Kiers et al., 2010). A potential consequence of such a weakening in mutualism strength is that the plants’ resilience to drought also decreases (Afkhami et al., 2014; Brunner et al., 2015; Márquez et al., 2007; Peay, 2016). Such a loss of drought resilience may increase the potential for arid grassland and shrubland ecosystems to degrade and collapse in the face of warming‐induced decreases in precipitation.

### 5.2 Tropical forests

Trees are the dominant habitat‐structuring organisms of forests (Ellison et al., 2005). Following scenario 1, trees in tropical regions modify the environment to their own benefit by outcompeting grasses that would otherwise facilitate wildfires that in turn promote open savannas or grasslands (Hirota et al., 2011). Moreover, in particularly large and/or dense forest patches, trees can generate a vegetation‐climate feedback in which the trees via evapotranspiration maintain a moist microclimate that stimulates rainfall, thereby stabilizing tree‐dominance and preventing grassland encroachment (Hirota et al., 2011; Lewis, 2006; Lindenmayer et al., 2016; Zemp et al., 2017).

Similar to arid ecosystems, tropical trees also commonly engage in endophytic mutualisms that, following scenario 3 in the model, can increase tree tolerance to drought and wildfires (Brunner et al., 2015). Simultaneously, following scenario 2, many tropical tree species engage in mutualisms that act on a second stressor—that is, reduced reproductive capacity—as they depend on pollinators and seed dispersers for their reproduction (Janzen & Martin, 1982; Peres et al., 2016; Rodriguez‐Cabal et al., 2007). Extirpation of monkeys, birds, bats, and other vital seed dispersers and pollinators, however, weaken the strength of these plant–animal mutualisms in many areas. In the Amazon, for instance, overhunting has severely reduced populations of seed‐dispersing vertebrates, causing “empty forests” (Redford, 1992). Consequently, seed dispersal becomes depressed, reducing tree recruitment and causing forest canopies to become more open (Peres et al., 2016). This can in turn weaken the tree‐microclimate feedback that mitigates the first stressor (drought), thus increasing the risk of forest collapse, particularly in many tropical regions where global warming is altering precipitation regimes.

### 5.3 Salt marshes

Salt‐tolerant marsh grasses are important foundation species along temperate and subtropical coastlines. By progressively baffling currents and waves with increasing shoot density and patch size, marsh grasses stabilize and elevate the sediment bed and increase nutrient availability (van Bouma et al., 2009; de Koppel et al., 2005; Temmerman et al., 2007). Following scenario 1, these self‐facilitative feedbacks have been found to increase ecosystem resistance to small‐scale disturbances, but also increase the potential for bistability and collapse following intense, large‐scale disturbances like winter storms (van van Belzen et al., 2017; de Koppel et al., 2005).

Along the US Atlantic and Gulf coasts, ribbed mussels (*Geukensia demissa*) aggregate in the mud around cordgrass stems, where they profit from stable settlement substrate and canopy shading (Altieri et al., 2007; Borst et al., 2018). In return, as mussels filter phytoplankton and clay particles from the water column, they deposit nutrient‐rich pseudofaeces, stimulating cordgrass growth and survival (Bertness, 1984). This mussel fertilization acts in concert with cordgrass particle trapping to alleviate nutrient limitation, following our model scenario 3.

In addition to enhancing nutrient availability, mussels can also enhance soil moisture and decrease salinity stress during hot dry spells, increasing cordgrass survival by 5–25 times (Angelini et al., 2016). During drought, the mutualism therefore buffers a second stressor in ways similar to scenario 2. Recent work, however, suggests that intense or repetitive droughts may ultimately exceed the mutualism's buffering capacity (Derksen‐Hooijberg et al., 2019). Should these extreme events increase in both severity and frequency as predicted, the salinity‐buffering mechanism will be under intensifying pressure, increasing the likelihood of salt marsh collapse (Angelini et al., 2016; Derksen‐Hooijberg et al., 2019).

### 5.4 Seagrass meadows

Seagrasses are habitat‐forming, flowering plants in shallow coastal areas worldwide (Larkum et al., 2006). Similar to salt marsh plants, dense and large seagrass meadows reduce hydrodynamic energy and trap suspended particles, while their root mats prevent sediment resuspension, increasing light penetration (Christianen et al., 2013; Hansen & Reidenbach, 2012; van der Heide et al., 2007; Koch, 2001). Following scenario 1, these habitat modifications increase seagrass growth and survival, but also increase the potential for bistability (van der Heide et al., 2007; Maxwell et al., 2017).

Although sediment trapping and stabilization stimulate seagrass growth, they also cause a negative feedback as organic matter from the water column accumulates in the sediment, and its anaerobic decomposition involving sulfate‐reducing bacteria has the potential to produce toxic levels of sulfides (de Fouw et al., 2016, 2018; van der Heide et al., 2012; Maxwell et al., 2017). Although seagrasses stimulate sulfide oxidation by releasing oxygen from their roots, sulfide production can outpace oxygen release under warmer conditions, resulting in sulfide accumulation and seagrass mortality (de Fouw et al., 2016, 2018). Following model scenario 2, over 90% of seagrasses growing in subtropical to tropical conditions, and over 50% in temperate areas, are associated with lucinid bivalve mutualists that have endosymbiotic sulfide‐oxidizing bacteria in their gills (van der Heide et al., 2012). In this pervasive facultative mutualism, the lucinid‐bacteria consortium profits from both the sulfide and released oxygen and, in consuming and oxidizing sulfide, alleviates sulfide toxicity stress experienced by seagrass (van der Heide et al., 2012).

Drought, however, was recently shown to disrupt this mutualism in West African intertidal seagrass meadows. On the mudflats of Banc d’Arguin, a drought in 2011 initiated seagrass degradation, decreasing oxygen release from the roots, and causing the mutualism to collapse. This, in turn, spiked sediment sulfide levels, amplifying seagrass die‐off and causing landscape‐scale degradation (de Fouw et al., 2016, 2018). These results illustrate that extreme conditions, such as drought or excessive eutrophication (Maxwell et al., 2017), may exceed the buffering capacity of this mutualism, thus triggering its breakdown and seagrass mass mortality. After such collapse, recovery may only be possible once sediment organic matter and sulfide levels have been dramatically reduced (de Fouw et al., 2018).

## 6 PERSPECTIVES

Collectively, our findings highlight that foundation species often facilitate both themselves and associated community members through density‐ or patch size‐dependent alterations of abiotic conditions, and that they commonly engage in facultative mutualistic interactions that initiate additional feedbacks. Our model simulations, supported by empirical observations from four different types of ecosystems, suggest that the self‐facilitative feedback can be amplified by the mutualistic feedback, increasing the potential for nonlinear ecosystem responses and bistability in the face of increasing human‐mediated global change stressors (Figure 3a). Specifically, our modeling results suggest that when the self‐facilitative and mutualistic feedbacks operate on the same environmental stressor, ecosystem resistance to stress can be particularly high, but, consequently, also the range of hysteresis and thus the risk of catastrophic collapse. Our real‐world examples highlight the relevance of these findings as they indicate that this may occur when (a) drought resistance is bolstered by both desert plants and their endophytes (Márquez et al., 2007; Peay, 2016), and (b) nutrient‐enhancement is sustained both by salt marsh grasses and ribbed mussels (Bertness, 1984). Although this “amplification effect” is less dramatic when the self‐facilitative and mutualistic feedbacks operate on different stressors, their simultaneous functioning can have important consequences for ecosystem resilience, as bistability may now be generated along two (instead of one) stress gradients (Figure 3a,b). In our real‐world examples, these dynamics appear to occur in tropical forest where trees engineer the microclimate to support their own persistence, and simultaneously benefit from a seed‐dispersing mutualist feedback.

**FIGURE 4:**
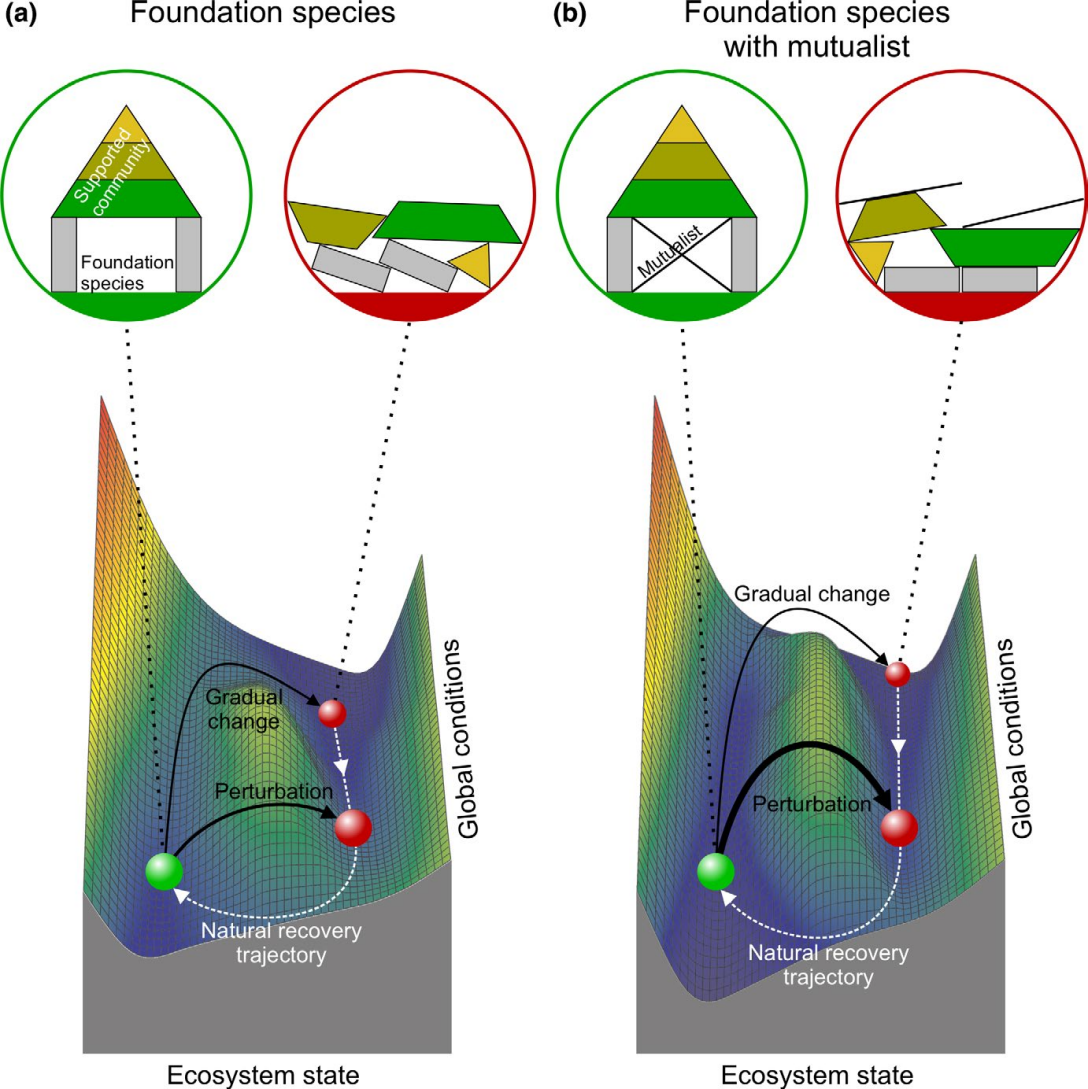
Stability landscape of ecosystems shaped by foundation species without (a) and with mutualists (b). Ecosystem A is controlled by a self‐facilitative feedback, and hence, a relatively small change in global conditions (or perturbation) is sufficient to cause the healthy (green) system to collapse (red). Contrastingly, as ecosystem B is controlled by self‐facilitative and mutualistic feedbacks that amplify each other, a more severe change in global conditions (or perturbation) is required for a collapse. If collapsed due to gradual changes, recovery requires conditions to be improved beyond the point of collapse, a pathway that is much longer for ecosystem B

These central findings build upon a number of prior studies demonstrating that mutualists can broaden species’ environmental tolerance ranges (e.g., Afkhami et al., 2014; Kiers et al., 2010). However, our work further suggests that when facultative mutualistic interactions involve foundation species, they increase both their resistance to gradual changes or sudden perturbations, and their propensity to exhibit nonlinear ecosystem responses to anthropogenic global change pressures (Figure 4). Thus, consideration of both self‐facilitative and mutualism‐generated feedbacks is likely to be essential for predicting the stress thresholds beyond which foundation species and their associated communities and ecosystem functions will collapse, as well as the level of environmental stress mitigation that must be achieved to trigger natural recovery.

More broadly, the results of our modeling and literature review emphasize the importance of acknowledging and quantifying how multiple feedbacks interact to drive ecosystem dynamics. Recent work from coral reefs and seagrass meadows has similarly highlighted that foundation species can be involved in multiple feedbacks that collectively amplify nonlinear responses (see Maxwell et al., 2017; van de Leemput et al., 2018). Moreover, the strength of such feedbacks and their level of interaction are likely highly context‐dependent, varying in strength along environmental gradients or in response to changing conditions (Maxwell et al., 2017), an area of study that requires far more research. Specifically, for ecosystems shaped by foundation species, it is important to identify those that are simultaneously engaged in self‐facilitative and facultative mutualistic feedbacks. Clearly, although our real‐world examples highlight only four ecosystems, there are many more of ecosystems with foundation species where both feedback types can occur and interact (see Table 1).

A vital next step is to resolve the relative strength of the self‐facilitative and facultative mutualistic feedbacks in modulating the dynamics of foundation species‐dominated ecosystems. A first approach could be to construct a more system‐specific simulation model to assess the potential for nonlinear behavior and bistability in response to increasing global stressors. A second possibility is to correlatively investigate the response of such ecosystems when they are undergoing a sudden perturbation. Recent examples were presented by de Fouw et al. (2016) and Angelini et al. (2016) where intertidal seagrass meadows with lucinid bivalves and salt marshes with ribbed mussels partly collapsed due to droughts. Although they do not provide definitive proof for bistability, new statistical techniques such as potential analysis may yield important clues regarding the importance of feedbacks in driving ecosystem dynamics (Dakos et al., 2015; de Fouw et al., 2016; Hirota et al., 2011; Scheffer et al., 2012).

The ultimate step is then to experimentally manipulate both the self‐facilitative and mutualistic feedbacks across relevant stress gradients to identify nonlinear responses and alternative stable states, and to test whether the mutualist or the foundation species is the weaker link when conditions change. To our knowledge, such elaborate experiments, which basically represent an empirical version of our model simulations, have not yet been conducted with foundation species and their mutualists. However, different parts of such an experiment have been carried out across a range of different ecosystem types. For instance, Afkhami et al. (2014) manipulated endophyte mutualisms across a range of environmental conditions using field and greenhouse experiments to empirically demonstrate mutualism‐mediated broadening of environmental tolerance to drought in plants. In addition, Angelini et al. (2016) experimentally demonstrated mutualism‐mediated drought resistance in US salt marshes during a heat spell. Neither study, however, simultaneously manipulated the strength of the self‐facilitative feedback (e.g., by manipulating plant density or patch size). Experiments in which both the foundation species and the mutualist were manipulated have been carried out with seagrasses and lucinids (van der Heide et al., 2012), and with cordgrass and ribbed mussels (Borst et al., 2018). In these cases, however, the environmental conditions were not manipulated. Moreover, none of the above experimental studies focused on identifying nonlinear responses or bistability across stress gradients such as presented in our model analyses, emphasizing that understanding these systems through experimental manipulation is currently an important caveat.

## 7 POTENTIAL MANAGEMENT IMPLICATIONS

From a conservation standpoint, it is of primary importance to identify whether foundation species generate self‐facilitative feedbacks, mutualistic feedbacks, or both, and to measure their strength. If feedbacks are indeed important, our work suggests that, ideally, managers and regulators should aim to maintain stress levels well below the point where these feedbacks become vital for foundation species persistence (i.e., <0.3 in our model; see Figure 3). Obviously, this may be infeasible, especially when a stressor is initiated by global rather than local processes, such as droughts or heat waves. In such cases, however, it may be possible to reduce local stressors for the purpose of increasing foundation species' capacity to persist under increasing global stress. Specifically, as suggested by our model and earlier work (He & Silliman, 2019; Scheffer et al., 2015), when selfacilitative and mutualistic feedbacks both buffer against the same global stressor (i.e., stressor 1), mitigation of a second local stressor that is not affected by the feedbacks (see Figure 3c, scenario 3) can be highly effective in enabling the ecosystem to persist in a foundation species‐dominated state. The underlying reason for this is that the maximum net growth of the foundation species increases linearly with a reduction of stressor 2, which in turn increases both self‐facilitation and mutualism feedback strength and thus the foundation species' capacity to buffer stressor 1. Furthermore, when one of these feedbacks instead buffers a local stressor, the response of the foundation species to local improvements, and therefore also its ability to withstand and mitigate the global stressor, becomes nonlinear.

Even when local stressors are mitigated via proactive management or regulation, continued global environmental change may ultimately cause foundation species to become fully reliant on their facultative mutualistic partners. Under such circumstances, further escalation of the global stress or sudden perturbations, such as extreme storms or consumer outbreaks, may ultimately exceed the buffering capacity of the self‐facilitative and/or mutualistic feedbacks, causing foundation species collapse. Once degraded, self‐facilitative feedbacks required for sustaining the foundation species are absent, yielding establishment thresholds that prevent natural recovery. Moreover, these dynamics may be exacerbated by the absence of mutualists that can help improve environmental conditions and the foundation species’ health (Angelini et al., 2016; Angelini & Silliman, 2012). Consequently, environmental conditions need to be improved much more than the level of stress that provoked the collapse to initiate natural, or unassisted, recovery (hysteresis) under such circumstances (Figure 4).

In the context of restoration or habitat creation efforts, our findings suggest that harnessing self‐facilitation and mutualisms can enhance the success of such interventions to regain foundation species and their ecological benefits (Gagnon et al., 2020; Valdez et al., 2020). Indeed, recent experimental work in salt marshes highlights that including self‐facilitation into restoration designs by clumping cordgrass transplants rather than planting them in dispersed arrays can double restoration yields (Silliman et al., 2015). Moreover, integrating mutualisms into restoration by co‐transplantation of cordgrass and mussels can enhance success by a similar margin (Borst et al., 2018). At the same time, however, it is important to realize that such reliance on self‐facilitation and mutualisms comes at the cost of increased threshold behavior, which decreases predictability and may unintentionally set systems up for sudden collapse in the long run.

## 8 CONCLUSIONS

It is clear that the biodiversity and functioning of many terrestrial, freshwater, and marine benthic ecosystems hinges on habitat‐forming foundation species (Angelini et al., 2011; Borst et al., 2018; Ellison, 2019). Such spatially dominant habitat‐forming organisms (e.g., trees, terrestrial shrubs and grasses, marine and freshwater macrophytes, bivalve and coral reefs) create complex biogenic structures that ameliorate physical stress and modulate resource availability. Although it is widely appreciated that associated species often benefit from such habitat modification, foundation species also facilitate their own growth through these same mechanisms. Although such self‐facilitative and mutualistic feedbacks can act as a buffer against increasing environmental stress, theory and observations suggest that when they are disrupted, foundation species can experience rapid mortality, resulting in persistent collapse of the ecosystem they support.

This study highlights that many foundation species engage in facultative mutualisms that, by providing reciprocal benefits, generate a second positive feedback that may act on the same or a different stressor as the self‐facilitative feedback. Overall, our model and case studies suggest that such mutualisms, which are pervasive in natural systems, pose a double‐edged sword in the face of human‐mediated global change. Specifically, mutualisms help protect and restore foundation species‐structured ecosystems in times of rapid, global environmental change, but reliance on self‐facilitative and mutualistic feedbacks may come at the inherent cost of increased threshold behavior, increasing the potential for bistability and sudden, persistent collapse.

## CONFLICT OF INTEREST

The authors declare no conflict of interest.

## AUTHOR CONTRIBUTION

**Tjisse van der Heide:** Conceptualization (equal); Formal analysis (lead); Investigation (equal); Writing‐original draft (lead); Writing‐review & editing (equal). **Christine Angelini:** Conceptualization (supporting); Formal analysis (supporting); Investigation (equal); Writing‐original draft (supporting); Writing‐review & editing (equal). **Jimmy De Fouw:** Conceptualization (supporting); Formal analysis (supporting); Investigation (equal); Writing‐original draft (supporting); Writing‐review & editing (equal). **Johan S. Eklöf:** Conceptualization (equal); Formal analysis (supporting); Investigation (equal); Writing‐original draft (supporting); Writing‐review & editing (equal).

## Data Availability

No new data were used.
